# Strain-induced high ferromagnetic transition temperature of MnAs epilayer grown on GaAs (110)

**DOI:** 10.1186/1556-276X-6-125

**Published:** 2011-02-09

**Authors:** Pengfa Xu, Jun Lu, Lin Chen, Shuai Yan, Haijuan Meng, Guoqiang Pan, Jianhua Zhao

**Affiliations:** 1 State Key Laboratory for Superlattices and Microstructures, Institute of Semiconductors, Chinese Academy of Sciences, P.O. Box 912, Beijing 100083, China; 2 National Synchrotron Radiation Laboratory, University of Science and Technology of China, Hefei 230029, China

## Abstract

MnAs films are grown on GaAs surfaces by molecular beam epitaxy. Specular and grazing incidence X-ray diffractions are used to study the influence of different strain states of MnAs/GaAs (110) and MnAs/GaAs (001) on the first-order magnetostructural phase transition. It comes out that the first-order magnetostructural phase transition temperature *T*_t_, at which the remnant magnetization becomes zero, is strongly affected by the strain constraint from different oriented GaAs substrates. Our results show an elevated *T*_t _of 350 K for MnAs films grown on GaAs (110) surface, which is attributed to the effect of strain constraint from different directions.

PACS: 68.35.Rh, 61.50.Ks, 81.15.Hi, 07.85.Qe

## Introduction

Today, there is growing interest for realization of new technologies utilizing spin degree of freedom of electrons in semiconductor devices [1]. The technology of manipulating spin in semiconductors promises devices with enhanced functionality and higher speed. A prerequisite for realization of such kind of devices is development of solid-state spin injectors at room temperature. Diluted magnetic semiconductors (DMSs) and ferromagnet/semiconductor hybrids are two important components for efficient spin injection. The exploitation of DMSs, however, is severely hindered by their low Curie temperature due to the low solubility of transition metals in semiconductors [2,3]. With room-temperature ferromagnetism and high crystal quality, MnAs has been epitaxied on (001)-, (110)-, (111)-, and (113)-oriented GaAs substrates [4-9]. Moreover, MnAs/GaAs having sharper interface than that of Fe/GaAs has been presented [10,11]; the sharp interface is considered to be crucial for obtaining higher transmission efficiencies. Recently, spin injection from MnAs into GaAs has been demonstrated [12], and the spin-dependent tunneling experiments show that the spin polarization at MnAs/GaAs interfaces is high [13,14]. Therefore, MnAs/GaAs hybrid is attracting more and more attention for its potential applications in spin injection, magnetic tunneling junctions, and magnetically logic devices.

The first-order magnetostructural phase transition is a long-standing topic in magnetism [15-20]. Bulk MnAs shows a coupled first-order magnetostructural phase transition from the ferromagnetic hexagonal α-phase (P6(3)/mmc) to the paramagnetic orthorhombic β-phase (Pnma) which is contracted in volume by 2% at about 318 K. For epitaxial MnAs films on GaAs substrate, the transition proceeds continuously over a broad temperature range with coexistence of the two phases. The phase coexistence results in a considerable fraction of MnAs epitaxial films which are usually in paramagnetic phase at ~30°C, a strong limitation for room temperature spintronic devices. The first-order magnetostructural phase transition temperature *T*_t_, at which the remnant magnetization becomes zero, can be enhanced either by applying an external magnetic field or by growing MnAs films on different oriented GaAs substrates [21]. For example, *T*_t _for MnAs films grown on GaAs (111)B is higher than that grown on GaAs (001) [18,22,23].

Epitaxial MnAs films on GaAs (001) and GaAs (111)B have been thoroughly investigated [4-6], while little attention has been paid to MnAs films grown on GaAs (110) [7]. The spin relaxation time is considered crucial important for practical application of spin memory devices or spin quantum computers. In GaAs (110) quantum wells, the spin relaxation time is in nanosecond range, much longer than that in GaAs (001) where the spin relaxation time is in picoseconds range [7]. More work is expected for investigation of MnAs films grown on GaAs (110) surfaces. In this work, we will present that epitaxial MnAs films grown on GaAs (110) are with a different strain states from MnAs films grown on GaAs (001), and α-phase can coexist with β-phase to a higher temperature (remnant magnetization becomes zero when the temperature reaches 350 K).

### Experimental procedure

The MnAs films were grown on GaAs (110) and GaAs (001) substrates by molecular beam epitaxy with a 12-keV reflection high-energy electron diffraction (RHEED) to monitor the growth process. Before growth of MnAs, a 100-nm GaAs buffer layer was grown to smoothen the surface. For MnAs films grown on GaAs (110), the buffer layer was grown at a lower substrate temperature *T*_s _= 400°C and higher As_4_/Ga beam equivalent pressure (BEP) ratio of 50, while for the buffer layer grown on GaAs (001), a standard procedure (*T*_S _= 560°C, As_4_/Ga = 12) was used. The growth parameters and thickness of MnAs films on GaAs (110) and GaAs (001) are shown in Table 1. During MnAs growth, the surface is (1 × 2) reconstructed. By analyzing RHEED patterns taken during growth of MnAs, we get the following epitaxial relationship: $\left(1\bar{1}00\right)$ MnAs//(110) GaAs, [0001] MnAs//[001] GaAs, and $\left(11\bar{2}0\right)$ MnAs//$[1\bar{1}0]\;$ GaAs. The streaky RHEED pattern becomes sharper after *in situ *cooling the sample from growth temperature to room temperature, indicating enhancement of crystal quality. The microstructure and interface of the MnAs films were characterized by high-resolution cross-sectional transmission electron microscopy (HRTEM), while the study of the magnetic domain structures was carried out by using magnetic force microscope (MFM). By using tapping/lift modes, the topographic and magnetic force images may be collected separately and simultaneously in the same area of the sample. The magnetic property of all the samples was measured by superconducting quantum interference device magnetometry with magnetic field parallel to the surface of samples. Anisotropic strain of the thin MnAs films was characterized by X-ray diffraction (XRD). The XRD experiments were performed at the U7B beam line of National Synchrotron Radiation Laboratory of China using a 0.154-nm wavelength monochromatic beam, which is selected through a double-crystal Si (111) monochromator, and triple-axis mode was used in these measurements in order to achieve high resolution. Strain information was obtained through measurements of in-plane and out-of-plane diffractions in the *ω*/2*θ *scan mode. Specially, grazing incidence geometry was performed for in-plane measurements (IP-GIXD).

**Table 1:** Growth parameters and the thickness for samples A-D

Sample	Growth temperature (°C)	As_4_/Mn BEP ratio	Furnace cooling	Thickness (nm)	GaAs substrate
A	230	300	N	11	GaAs (001)

B	210	175	N	3	GaAs (110)

C	210	300	N	11	GaAs (110)

D	210	175	Y	11	GaAs (110)

## Results and discussion

**Figure 1:**
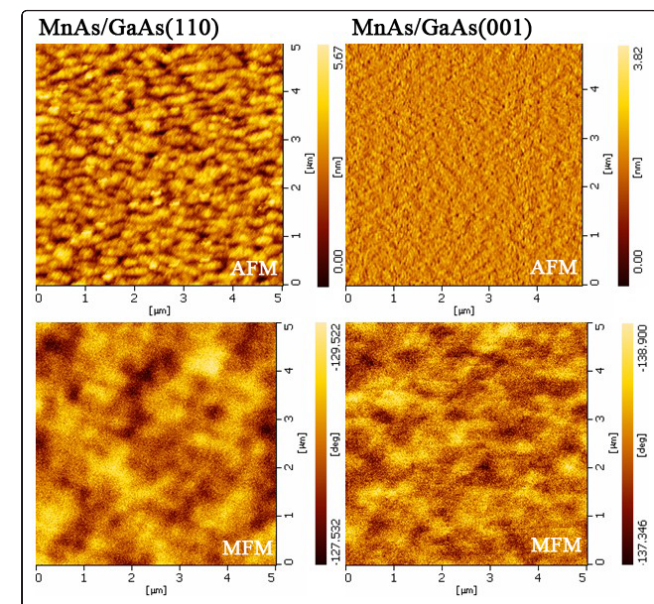
**Images of room-temperature AFM and MFM**. Room-temperature AFM (upper panel) and MFM (lower panel) images for 11-nm MnAs films grown on GaAs (110) (left) and GaAs (001) (right), taken from the growth surface.

**Figure 2:**
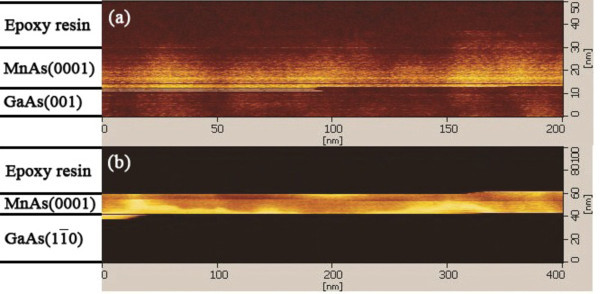
**Cross-sectional MFM images for (a) MnAs/GaAs (110) and (b) MnAs/GaAs (001)**.

**Figure 3:**
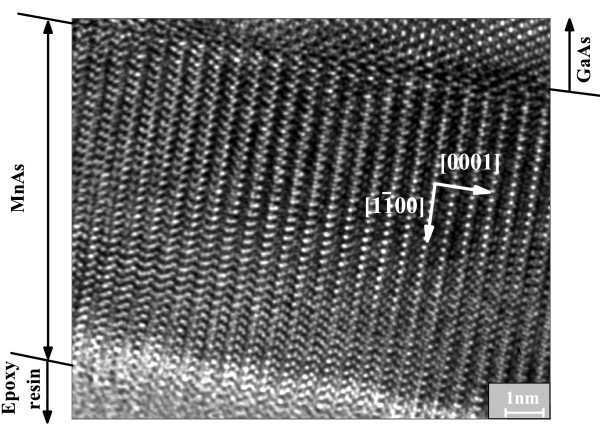
**HRTEM image of sample D (MnAs/GaAs (110))**. The crystallographic directions of the epitaxial film were indicated with white arrows.

Atomic force microscopy (AFM) and MFM images taken from the growth surface are shown in Figure 1. One can see from Figure 1 that there is no evident stripe pattern in AFM images. Generally, all the samples in this study are very thin, and the stripe height is roughly 1% of the film thickness. As shown in Figure 1, the magnetic domains are randomly distributed in MFM images for MnAs films grown on both GaAs (110) and GaAs (001). We also observed the cross-sectional MFM images for MnAs/GaAs (001) and MnAs/GaAs (110). Although we can see sharp interfaces between MnAs and GaAs from Figure 2a,b, we cannot see evident borders between ferromagnetic α-phase and paramagnetic β-phase, indicating that the two phases are mixing together. Our results are different from observations in a 200-nm MnAs film epitaxied on GaAs (001) presented in [24], in which ferromagnetic α-phase and paramagnetic β-phase are obviously separated. We assumed this phenomenon resulted from the too thin thickness of MnAs layer. Figure 3 shows the HRTEM image of sample D, MnAs/GaAs (110), from which we can observe that MnAs films have a well-ordered crystal orientation and a sharp interface between MnAs and GaAs. Judged from the chromatic aberration of MnAs and GaAs substrate, the thickness of the epitaxial MnAs film is 11 nm.

**Figure 4:**
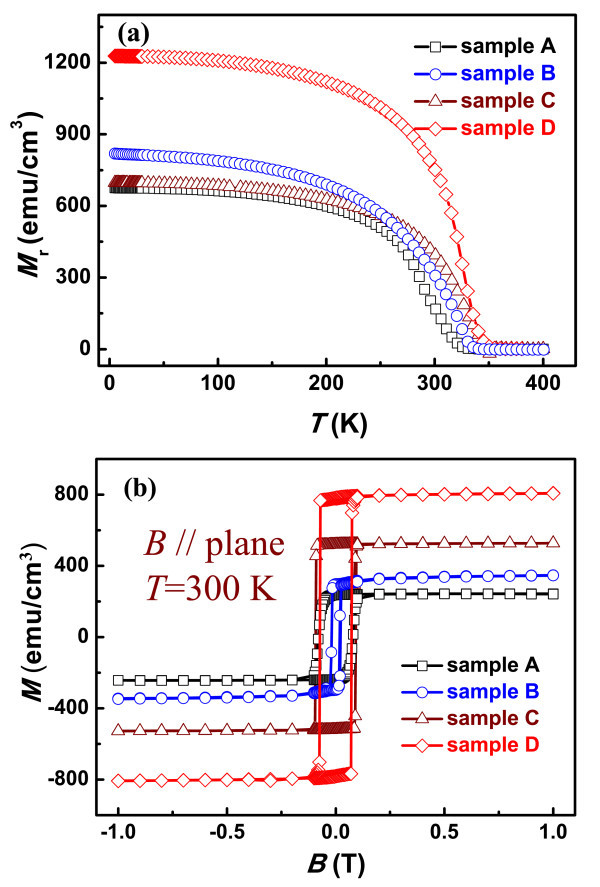
**Temperature dependence and magnetic field dependence of magnetization**. a Temperature dependence of the remnant magnetization *M*_r _for samples A-D. *M*_r _remains over zero even when the temperature reaches 340 and 350 K for samples B and D, respectively. **b **The magnetic field dependence of magnetization for MnAs grown on GaAs (110) and GaAs (001) at 300 K, under a magnetic field applied along the easy axis of magnetization.

The remnant magnetization *M*_r _as a function of temperature *T *is plotted in Figure 4a. The linear decrease of *M*_r _at low temperature is caused by thermal fluctuation, while the rapid decreasing at high temperature is caused by structural transition from hexagonal phase to orthorhombic phase. As the thickness can change magnetic property of MnAs epilayer [7,25], *M*_r _becomes zero when the temperature reaches 340 and 350 K for samples B and sample D, respectively. In accordance with RHEED pattern analysis given above, *M*_r _exceeds 1,200 emu/cm^3 ^at 5 K for sample D, which is a bit larger than the saturation magnetization reported for MnAs/GaAs (001) at 10 K with little crystal defect and optimum intra- and inter-stripe magnetic coupling [26]. The remarkable magnetic property difference between sample C and sample D may originate from the different growth conditions, such as the low substrate temperature and over pressure of As_4 _for sample C, or different stoichiometry. Figure 4b shows *M-H *hysteresis loops measured at room temperature with magnetic field applied along the direction of MnAs $\left(11\bar{2}0\right)$, the easy axis of magnetization. The magnetization hysteresis loops show a perfect square form for all the samples studied here.

**Figure 5:**
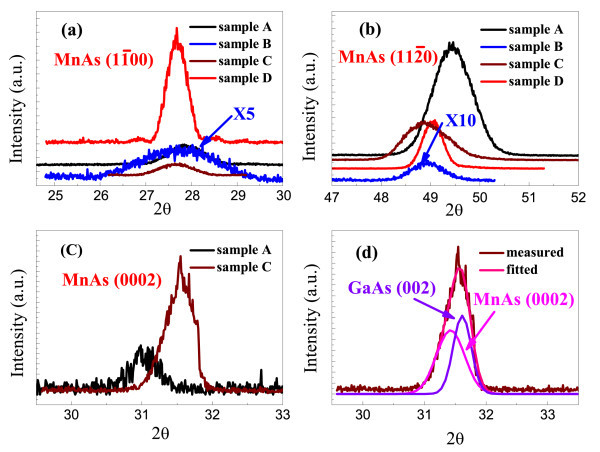
**XRD patterns**. XRD patterns measured by synchrotron radiation for reflections of MnAs $\left(1\bar{1}00\right)$ in the specular geometry, $\left(11\bar{2}0\right)$ and (0002) in the grazing incidence geometry for samples A (black), B (blue), C (wine), and D (red). The radial scan along MnAs (0002) of sample C can be fitted well by two peaks centered at 31.44 and 31.61 which can be ascribed to MnAs (0002) and GaAs (002), respectively (d).

In order to probe the effect of anisotropic strain on the first-order magnetostructural phase transition, we performed synchrotron XRD measurements. The experimental results are shown in Figure 5. The orthorhombic notation is used for the α-phase lattice parameters, in which *a*_ortho_, *b*_ortho_, and *c*_ortho _stand for the spacing between MnAs (0001), MnAs $\left(11\bar{2}0\right)$, and MnAs $\left(1\bar{1}00\right)$ , respectively. The lattice parameters, primitive cell volume, and transition temperature are shown in Table 2. Early in the 1960s, Bean and Rodbell and Menyuk et al. concluded that *T*_t _is proportional to the primitive cell volume and a larger primitive cell volume (*V*) corresponds to a higher *T*_t _based on the magnetostrictive model [15,16]. Clearly our experimental results cannot be explained by a simple effect of primitive cell volume variation, and *T*_t _is not a linear function of *V*. For example, as to all the epitaxial films studied here, the primitive cell volume is smaller than that of the bulk material, while ferromagnetic hexagonal α-phase can coexist with paramagnetic orthorhombic β-phase to a higher temperature. Furthermore, there is remarkable difference between lattice parameters for MnAs films grown on GaAs (001) and GaAs (110). For sample A, grown on GaAs (001), *a*_ortho _is larger, while *b*_ortho _and *c*_ortho _are smaller than that for sample D, grown on GaAs (110). In good agreement with the experimental and theoretical results of Iikawa et al. [23], all these changes result in a lower transition temperature (stretching of the lattice parameters in the basal plane results in a higher *T*_t_, while stretching of lattice parameters along the perpendicular direction lowers *T*_t_).

**Table 2:** Lattice parameters, primitive cell volume, and transition temperature of samples A-D and MnAs bulk [27]

	MnAs bulk	Sample A	Sample B	Sample C	Sample D
*a *(Å)	5.71	5.78	**5.71**	5.67	**5.69**

*b *(Å)	3.72	3.69	3.73	3.72	3.72

*c *(Å)	6.45	6.41	6.43	6.45	6.45

*T*_t _(Å)	313	325	335	350	350

*V *(Å^3^)	137.06	136.71	136.95	136.05	136.53

The lattice parameters shown in boldface were calculated from elastic constant tensor of MnAs.

### Summary

In summary, we have shown that the ferromagnetic order in MnAs can be extended to higher temperature by growing MnAs on GaAs (110). Ferromagnetic α-phase can coexist with paramagnetic β-phase to 350 K. By XRD measurements, it is found that *T*_t _is not a simple function of primitive cell volume, and stretching of lattice parameters in the basal plane or compressing of lattice parameter in the perpendicular direction results in a higher *T*_t_. The result described here attests to a strong link between anisotropic strain and epilayer properties. Understanding and mastering these characterizations may open a possibility to control magnetic properties via selection of substrate orientation and provide new possibilities for using MnAs epilayer in spintronic devices.

## Competing interests

The authors declare that they have no competing interests.

## Authors' contributions

PX, JL, LC, SY, HM carried out the sample preparation. PX, JL and GP participated in the XRD Measurements. PX carried out the MFM and SQUID measurements, the statistical analysis and drafted the manuscript. JZ conceived of the study and participated in its design and coordination. All authors read and approved the final manuscript.
